# Supplementary material for: The Small RNA Universe of Capitella teleta
Source: Front Mol Biosci. 2022 Feb 25;9:802814. doi: 10.3389/fmolb.2022.802814 (PMC8915122; doi:10.3389/fmolb.2022.802814)
Supplement: Supplementary file 1 [file DataSheet1.ZIP › Supplement/known/CAPTEscaffold_933_30847.pdf]

Provisional ID	:	CAPTEscaffold_933_30847
Score total	:	197969.7
Score for star read(s)	:	3.9
Score for read counts	:	197959.6
Score for mfe	:	1.6
Score for randfold	:	1.6
Score for cons. seed	:	3
Total read count	:	388301
Mature read count	:	385848
Loop read count	:	0
Star read count	:	2453

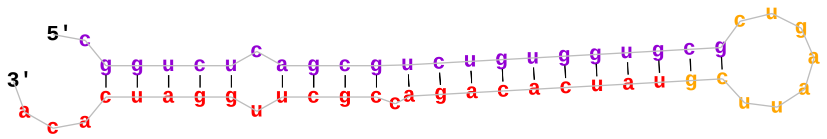

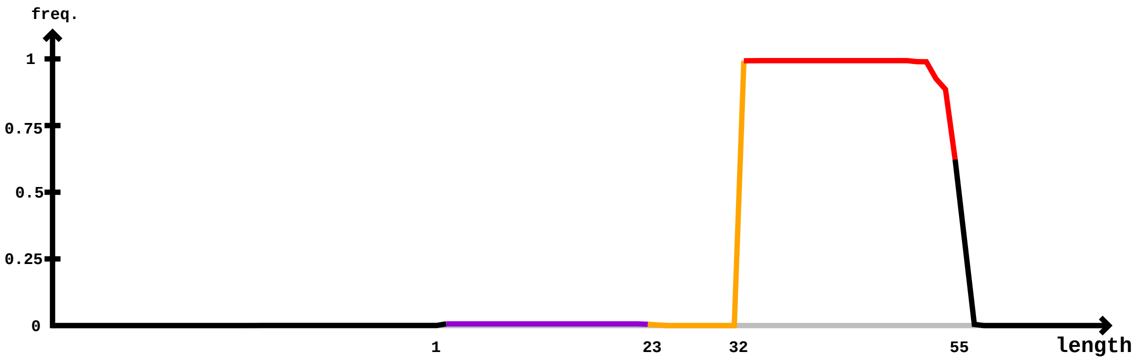

Star **Mature**

```
5'- cugccuccuucaccacccccuccugugcauggcucaccgcggucucagcgucuguggugcgcugaauucguaucacagaccgcuuggaucacagugcgcuguugugau  -3'  obs
    cugccuccuucaccacccccuccugugcauggcucaccgcggucucagcgucuguggugcgcugaauucguaucacagaccgcuuggaucacagugcgcuguugugau        exp
    cugccuccuucaccacccccuccugugcauggcucaccgcggucucagcgucuguggugcgcugaauucguaucacagaccgcuuggaucacagugcgcuguugugau        known
    .........(((((((........))).(((((.(((.(.(((((.(((((((((((((((.......))))))))))).)))).))))).).))).))))).)))).  reads   mm   sample

    ........uucaccacccccuccugugcauggcucaccg.....................................................................  1       0    seq
    ..............acccccuccugugcauggcucaccg.....................................................................  1       0    seq
    .................cccuccugugcauggcucaccg.....................................................................  3       0    seq
    ..................ccuccugugcauggcucaccg.....................................................................  6       0    seq
    ..................ccuccugugcauggcucaccgA....................................................................  1       1    seq
    ...................cucUugugcauggcucaccg.....................................................................  1       1    seq
    ...................cuccugugcauggcucaccg.....................................................................  24      0    seq
    ....................uccugugcauggcucacc......................................................................  1       0    seq
    ....................uccugugcauggcucaccg.....................................................................  60      0    seq
    .....................ccugugcauggcucaccg.....................................................................  5       0    seq
    .......................................cggucucagcgucuguggu..................................................  1       0    seq
    .......................................cggucucagcgucuguggug.................................................  5       0    seq
    .......................................cggucucagcUucuguggugc................................................  1       1    seq
    .......................................cggucucagAgucuguggugc................................................  2       1    seq
    .......................................cggucucagcgCcuguggugc................................................  1       1    seq
    .......................................cgguGucagcgucuguggugc................................................  1       1    seq
    .......................................cggucucagcgucuguggugG................................................  2       1    seq
    .......................................cggucucagcgucuguggugc................................................  622     0    seq
    .......................................cggucucagcgucugAggugc................................................  1       1    seq
    .......................................cAgucucagcgucuguggugc................................................  1       1    seq
    .......................................cggucucagcgucuguggugcg...............................................  1679    0    seq
    .......................................cggucucagcgucuguggugcC...............................................  2       1    seq
    .......................................cggucucGgcgucuguggugcg...............................................  1       1    seq
    .......................................cNgucucagcgucuguggugcg...............................................  1       1    seq
    .......................................cggucucagcgucugCggugcg...............................................  2       1    seq
    .......................................cggCcucagcgucuguggugcg...............................................  1       1    seq
    .......................................cggucucagcgucuguggCgcg...............................................  1       1    seq
    .......................................cggucucUgcgucuguggugcg...............................................  1       1    seq
    .......................................cggucucagcgucuguggugcA...............................................  1       1    seq
    .......................................cggucucagAgucuguggugcg...............................................  1       1    seq
    .......................................cggAcucagcgucuguggugcg...............................................  2       1    seq
```

```
                                       Star                           Mature
cugccuccuucaccacccccuccugugcauggcucaccgcggucucagcgucuguggugcgcugaauucguaucacagaccgcuuggaucacagugcgcuguugugau
.......................................cggucucagcgucugugAugcg...............................................	1	1	seq
.......................................cggucucaUcgucuguggugcg...............................................	1	1	seq
.......................................cggucucagcgucuguAgugcg...............................................	1	1	seq
.......................................Aggucucagcgucuguggugcg...............................................	1	1	seq
.......................................cggucucagcguUuguggugcg...............................................	1	1	seq
.......................................Uggucucagcgucuguggugcg...............................................	2	1	seq
.......................................cggucucagcgucuguCgugcg...............................................	1	1	seq
.......................................cggucucagcgucuguggugcU...............................................	86	1	seq
.......................................cggucucagcgucuguggugcgU..............................................	19	1	seq
.......................................cggucucagcgucuguggugcgc..............................................	1	0	seq
.......................................cggucucagcgucuguggugcgUu.............................................	3	1	seq
.......................................cggucucagcgucuguggugcgcugaauucg......................................	1	0	seq
........................................ggucucagcgucuguggugc................................................	1	0	seq
........................................ggucucagcgucuguggugcg...............................................	2	0	seq
.........................................gucucagcgucuguggugcg...............................................	3	0	seq
..........................................ucucagcgucuguggugc................................................	1	0	seq
.....................................................................Uuaucacagaccgcuugga....................	5	1	seq
.....................................................................Auaucacagaccgcuuggaucac................	1	1	seq
.....................................................................Auaucacagaccgcuuggaucaca...............	1	1	seq
.....................................................................Uuaucacagaccgcuuggaucaca...............	7	1	seq
......................................................................uUucacagaccgcuugga....................	1	1	seq
......................................................................uaucacagaccgcuuggG....................	2	1	seq
......................................................................uaucacagacAgcuugga....................	4	1	seq
......................................................................uaAcacagaccgcuugga....................	3	1	seq
......................................................................Aaucacagaccgcuugga....................	1	1	seq
......................................................................uaucaAagaccgcuugga....................	1	1	seq
......................................................................Caucacagaccgcuugga....................	1	1	seq
......................................................................uaucacagaccgcuugCa....................	1	1	seq
......................................................................uaucacagUccgcuugga....................	1	1	seq
......................................................................uaucaUagaccgcuugga....................	1	1	seq
......................................................................uaucacagaccgcuuAga....................	6	1	seq
......................................................................uaucGcagaccgcuugga....................	1	1	seq
......................................................................uaucacagaccgcuugAa....................	1	1	seq
......................................................................uGucacagaccgcuugga....................	1	1	seq
......................................................................uaucacagaccgAuugga....................	1	1	seq
......................................................................uaucacagaccgcuugga....................	1465	0	seq
......................................................................uaucacagaccgcuuCga....................	1	1	seq
......................................................................Aaucacagaccgcuuggau...................	1	1	seq
......................................................................uaucacagaccgcuuggaA...................	1	1	seq
......................................................................uaucacagaccgcuuggau...................	165	0	seq
......................................................................uaucacagaccgcuuAgau...................	2	1	seq
......................................................................uaucacagaccgcuuggaG...................	2	1	seq
......................................................................uaucacagaGcgcuuggauc..................	2	1	seq
......................................................................uGucacagaccgcuuggauc..................	5	1	seq
......................................................................uaucacagaccgcGuggauc..................	3	1	seq
......................................................................Caucacagaccgcuuggauc..................	3	1	seq
......................................................................uaucacagaccgcuuggauA..................	7	1	seq
......................................................................uaucaUagaccgcuuggauc..................	1	1	seq
......................................................................uNucacagaccgcuuggauc..................	2	1	seq
......................................................................uaucGcagaccgcuuggauc..................	3	1	seq
......................................................................uaucacUgaccgcuuggauc..................	3	1	seq
......................................................................uauAacagaccgcuuggauc..................	9	1	seq
......................................................................uUucacagaccgcuuggauc..................	2	1	seq
......................................................................uaucacagaAcgcuuggauc..................	7	1	seq
......................................................................uaucacagUccgcuuggauc..................	1	1	seq
......................................................................uaucacagaccgcAuggauc..................	4	1	seq
......................................................................uaucacagaccgcuugUauc..................	2	1	seq
......................................................................uaucacagaccgAuuggauc..................	7	1	seq
......................................................................uaucacagaUcgcuuggauc..................	3	1	seq
......................................................................uaucacagaccgcCuggauc..................	5	1	seq
......................................................................uaucUcagaccgcuuggauc..................	3	1	seq
......................................................................uaucacagaccgcuCggauc..................	3	1	seq
......................................................................uaucacagacGgcuuggauc..................	2	1	seq
......................................................................uaucacagaccgcuuUgauc..................	3	1	seq
......................................................................uaucacagaccgcuuggaAc..................	5	1	seq
......................................................................uaucacagaccgcuugAauc..................	1	1	seq
......................................................................Gaucacagaccgcuuggauc..................	7	1	seq
......................................................................uaucacagGccgcuuggauc..................	8	1	seq
......................................................................uaucacaUaccgcuuggauc..................	2	1	seq
......................................................................uaucacaAaccgcuuggauc..................	5	1	seq
```

Star **Mature**

cugccuccuucaccacccccuccugugcauggcucaccgcggucucagcgucuguggugcgcugaauucguaucacagaccgcuuggaucacagugcgcuguugugau			
....uaucacagacUgcuuggauc..................	11	1	seq
....uaucacagaccgcuuggauc..................	24120	0	seq
....uaCcacagaccgcuuggauc..................	2	1	seq
....Naucacagaccgcuuggauc..................	6	1	seq
....uaucacGgaccgcuuggauc..................	6	1	seq
....uaucacagaccgcuuggauU..................	8	1	seq
....uaucacagaccUcuuggauc..................	1	1	seq
....uaucacagaccgcuuggaCc..................	1	1	seq
....uaucacagaccgcuuCgauc..................	6	1	seq
....uaucaNagaccgcuuggauc..................	1	1	seq
....uaucacagaccgcuuggCuc..................	1	1	seq
....uaucacagaccgUuuggauc..................	6	1	seq
....uaucaAagaccgcuuggauc..................	13	1	seq
....uaucacagaccgcuAggauc..................	7	1	seq
....uaucacagaccgcuuggGuc..................	2	1	seq
....uaucacagacNgcuuggauc..................	1	1	seq
....uaucacagacAgcuuggauc..................	18	1	seq
....uaucacagaccgcuugCauc..................	1	1	seq
....uaucacagCccgcuuggauc..................	16	1	seq
....uaucacagaccgcuuAgauc..................	47	1	seq
....Aaucacagaccgcuuggauc..................	75	1	seq
....uauUacagaccgcuuggauc..................	11	1	seq
....uaucaGagaccgcuuggauc..................	1	1	seq
....uaAcacagaccgcuuggauc..................	33	1	seq
....uaucacagaccCcuuggauc..................	2	1	seq
....uaucacagUccgcuuggauca.................	3	1	seq
....uaucacagaccgcuuggauGa.................	1	1	seq
....uaucacagaccgcuuAgauca.................	38	1	seq
....uaucacagaccgcuuggaGca.................	1	1	seq
....uaucacagCccgcuuggauca.................	8	1	seq
....uaucacagGccgcuuggauca.................	1	1	seq
....uaucacagaccgcuuggauca.................	15476	0	seq
....uaucaUagaccgcuuggauca.................	2	1	seq
....uaucacaAaccgcuuggauca.................	3	1	seq
....uaucacagaccgcuugAauca.................	1	1	seq
....uaucacGgaccgcuuggauca.................	2	1	seq
....uaucacagaccgcuuUgauca.................	1	1	seq
....Naucacagaccgcuuggauca.................	4	1	seq
....Gaucacagaccgcuuggauca.................	3	1	seq
....uaucacagaccgcuuggauAa.................	6	1	seq
....uaucacagaccgcuuggauUa.................	6	1	seq
....uaucacagaccgcuuggaAca.................	5	1	seq
....uaucacagaccgGuuggauca.................	3	1	seq
....uaucacagaccgcuGggauca.................	1	1	seq
....uaucacagaccgcuAggauca.................	4	1	seq
....uaucacagacUgcuuggauca.................	6	1	seq
....uaucacaCaccgcuuggauca.................	1	1	seq
....uaucacagaccgcuuggaucU.................	24	1	seq
....uaucacagaccgcuuggGuca.................	3	1	seq
....uaucaAagaccgcuuggauca.................	7	1	seq
....uNucacagaccgcuuggauca.................	1	1	seq
....uaucacagaccgcuCggauca.................	5	1	seq
....uaucacagaccAcuuggauca.................	3	1	seq
....uaucacagaccgcAuggauca.................	2	1	seq
....uauAacagaccgcuuggauca.................	6	1	seq
....uaucacUgaccgcuuggauca.................	1	1	seq
....Aaucacagaccgcuuggauca.................	50	1	seq
....uaucacagaccgcuuggaCca.................	1	1	seq
....uaucGcagaccgcuuggauca.................	3	1	seq
....uaucUcagaccgcuuggauca.................	3	1	seq
....uaucacagaccUcuuggauca.................	1	1	seq
....uGucacagaccgcuuggauca.................	1	1	seq
....uaCcacagaccgcuuggauca.................	1	1	seq
....uaucCcagaccgcuuggauca.................	1	1	seq
....uaAcacagaccgcuuggauca.................	14	1	seq
....uaucacagaccgAuuggauca.................	1	1	seq
....uaucacagaAcgcuuggauca.................	4	1	seq
....uaucacagaUcgcuuggauca.................	2	1	seq
....uaucacagaccgcuuggUuca.................	3	1	seq
....uaucacagaccgcuuggaucG.................	27	1	seq

Star Mature

```
cugccuccuucaccacccccuccugugcauggcucaccgcggucucagcgucuguggugcgcugaauucguaucacagaccgcuuggaucacagugcgcuguugugau
......................................................................uaucacagaccCcuuggauca.................	1	1	seq
......................................................................uaucacagaccgcuuCgauca.................	4	1	seq
......................................................................uaucacagacAgcuuggauca.................	9	1	seq
......................................................................uaucacagaccgcuugAaucac................	13	1	seq
......................................................................uaucacaAaccgcuuggaucac................	10	1	seq
......................................................................uaucacagaccgcuuggCucac................	3	1	seq
......................................................................uaucacagaccAcuuggaucac................	10	1	seq
......................................................................uaucacaUaccgcuuggaucac................	7	1	seq
......................................................................uaucacGgaccgcuuggaucac................	11	1	seq
......................................................................uaucacagacUgcuuggaucac................	80	1	seq
......................................................................uaucacaCaccgcuuggaucac................	10	1	seq
......................................................................uaucacagaccgUuuggaucac................	4	1	seq
......................................................................uaucacagaccgcuuggUucac................	17	1	seq
......................................................................uaucacCgaccgcuuggaucac................	3	1	seq
......................................................................uaucacagaccgcuuUgaucac................	9	1	seq
......................................................................uaucGcagaccgcuuggaucac................	21	1	seq
......................................................................uaucaNagaccgcuuggaucac................	3	1	seq
......................................................................Gaucacagaccgcuuggaucac................	36	1	seq
......................................................................uaucacagaAcgcuuggaucac................	23	1	seq
......................................................................uaNcacagaccgcuuggaucac................	2	1	seq
......................................................................uaucacagaccgcuuggaucGc................	19	1	seq
......................................................................Aaucacagaccgcuuggaucac................	336	1	seq
......................................................................uaAcacagaccgcuuggaucac................	132	1	seq
......................................................................uaucacagaccgcuuggaucaG................	2	1	seq
......................................................................uaucacagaGcgcuuggaucac................	5	1	seq
......................................................................uauGacagaccgcuuggaucac................	2	1	seq
......................................................................uaucacagaccgcuuggaucac................	99968	0	seq
......................................................................uaucaAagaccgcuuggaucac................	52	1	seq
......................................................................uaucacagacAgcuuggaucac................	71	1	seq
......................................................................uaucacagaccgcuuggauUac................	55	1	seq
......................................................................uUucacagaccgcuuggaucac................	10	1	seq
......................................................................uaucacagaccgcuuggaucaU................	25	1	seq
......................................................................uaucacagaccCcuuggaucac................	3	1	seq
......................................................................uaucacUgaccgcuuggaucac................	2	1	seq
......................................................................uaucacagaccgcuuggaCcac................	13	1	seq
......................................................................uaucacagCccgcuuggaucac................	64	1	seq
......................................................................uaucacagaccgcuuggaucaA................	168	1	seq
......................................................................uaucacagaccgcuCggaucac................	21	1	seq
......................................................................uaucacagaUcgcuuggaucac................	16	1	seq
......................................................................uaucacagaccgcuuggauAac................	29	1	seq
......................................................................uaucacagUccgcuuggaucac................	13	1	seq
......................................................................Caucacagaccgcuuggaucac................	10	1	seq
......................................................................uaucacagaccgcAuggaucac................	15	1	seq
......................................................................uaucacagaccUcuuggaucac................	7	1	seq
......................................................................uaucacagaccgcuuCgaucac................	13	1	seq
......................................................................uaucaUagaccgcuuggaucac................	12	1	seq
......................................................................uaucaGagaccgcuuggaucac................	7	1	seq
......................................................................uNucacagaccgcuuggaucac................	4	1	seq
......................................................................uaucacagacGgcuuggaucac................	8	1	seq
......................................................................uaucacagaccgcuuggaucCc................	2	1	seq
......................................................................uaucacNgaccgcuuggaucac................	1	1	seq
......................................................................uauUacagaccgcuuggaucac................	53	1	seq
......................................................................uaucacagaccgcuuggauGac................	3	1	seq
......................................................................uaucacagaccgcuuggaAcac................	31	1	seq
......................................................................uGucacagaccgcuuggaucac................	11	1	seq
......................................................................uauAacagaccgcuuggaucac................	38	1	seq
......................................................................uaucacagaccgGuuggaucac................	6	1	seq
......................................................................uaucacagaccgcuugCaucac................	11	1	seq
......................................................................uaucUcagaccgcuuggaucac................	17	1	seq
......................................................................uaGcacagaccgcuuggaucac................	2	1	seq
......................................................................uaucacagaccgcuuggGucac................	12	1	seq
......................................................................uaucacagaccgcuuAgaucac................	239	1	seq
......................................................................uaucacagaccgAuuggaucac................	21	1	seq
......................................................................Naucacagaccgcuuggaucac................	30	1	seq
......................................................................uaucacagaccgcuGggaucac................	3	1	seq
......................................................................uaucacagaccgcuAggaucac................	43	1	seq
......................................................................uaucacagaccgcGuggaucac................	9	1	seq
......................................................................uaucacagaccgcuuggaucUc................	14	1	seq
......................................................................uaucacagGccgcuuggaucac................	18	1	seq
......................................................................uaCcacagaccgcuuggaucac................	11	1	seq
```

Star Mature

```
cugccuccuucaccacccccuccugugcauggcucaccgcggucucagcgucuguggugcgcugaauucguaucacagaccgcuuggaucacagugcgcuguugugau
......................................................................uaucacagaccgcuuggaGcac................   1      1   seq
......................................................................uaucacagaccgcuugUaucac................   9      1   seq
......................................................................uaucacagaccgcCuggaucac................   17     1   seq
......................................................................uaAcacagaccgcuuggaucaca...............   228    1   seq
......................................................................uaucacagCccgcuuggaucaca...............   125    1   seq
......................................................................uaucacagaccgcuuggaucGca...............   41     1   seq
......................................................................uaucacUgaccgcuuggaucaca...............   4      1   seq
......................................................................uaucacagaccgcuuggaGcaca...............   10     1   seq
......................................................................uaucacagaccgcuuCgaucaca...............   34     1   seq
......................................................................uaucacagaccgcuuggaucacC...............   241    1   seq
......................................................................uaucacagaccgcuugNaucaca...............   1      1   seq
......................................................................uaucacagaccgcuCggaucaca...............   31     1   seq
......................................................................uaucaAagaccgcuuggaucaca...............   87     1   seq
......................................................................uaucacagaccgcuuggaucacU...............   23188  1   seq
......................................................................uaucacagaccgcuuggaAcaca...............   51     1   seq
......................................................................uaucacagaccgcuuggauGaca...............   8      1   seq
......................................................................uUucacagaccgcuuggaucaca...............   25     1   seq
......................................................................uaucaNagaccgcuuggaucaca...............   4      1   seq
......................................................................uaucaUagaccgcuuggaucaca...............   24     1   seq
......................................................................uaucacGgaccgcuuggaucaca...............   34     1   seq
......................................................................uGucacagaccgcuuggaucaca...............   36     1   seq
......................................................................uaucacagacUgcuuggaucaca...............   114    1   seq
......................................................................uaucacagaccgcuuggaucaNa...............   1      1   seq
......................................................................uaucGcagaccgcuuggaucaca...............   46     1   seq
......................................................................uaGcacagaccgcuuggaucaca...............   1      1   seq
......................................................................uaucacagaccgcuuggUucaca...............   58     1   seq
......................................................................uaucacCgaccgcuuggaucaca...............   4      1   seq
......................................................................uauGacagaccgcuuggaucaca...............   10     1   seq
......................................................................uaucacagaccgcuuggCucaca...............   1      1   seq
......................................................................uaucacagaccgcCuggaucaca...............   24     1   seq
......................................................................uaucacagaccAcuuggaucaca...............   25     1   seq
......................................................................uaucacagaccgcuuggGucaca...............   55     1   seq
......................................................................uaucacaCaccgcuuggaucaca...............   20     1   seq
......................................................................uaucacagaccgcuuAgaucaca...............   570    1   seq
......................................................................uaucacagaccUcuuggaucaca...............   18     1   seq
......................................................................uaucacagaccgUuuggaucaca...............   15     1   seq
......................................................................uaucaGagaccgcuuggaucaca...............   10     1   seq
......................................................................uaucacagaUcgcuuggaucaca...............   28     1   seq
......................................................................uaucacaUaccgcuuggaucaca...............   11     1   seq
......................................................................uaCcacagaccgcuuggaucaca...............   16     1   seq
......................................................................uaucacagaccgcuugUaucaca...............   22     1   seq
......................................................................uaucacagaAcgcuuggaucaca...............   53     1   seq
......................................................................uaucacagGccgcuuggaucaca...............   29     1   seq
......................................................................uaucacagaccgcuuggaucaGa...............   14     1   seq
......................................................................uaucacagUccgcuuggaucaca...............   17     1   seq
......................................................................Gaucacagaccgcuuggaucaca...............   68     1   seq
......................................................................uaucacagaccgcuuggaucaUa...............   30     1   seq
......................................................................Caucacagaccgcuuggaucaca...............   29     1   seq
......................................................................uaucacagaccgcuuggaucUca...............   32     1   seq
......................................................................uNucacagaccgcuuggaucaca...............   4      1   seq
......................................................................uauAacagaccgcuuggaucaca...............   61     1   seq
......................................................................uaucacagacAgcuuggaucaca...............   174    1   seq
......................................................................uaucacagaccgGuuggaucaca...............   15     1   seq
......................................................................uaucacagaccCcuuggaucaca...............   7      1   seq
......................................................................uaucacagaccgcuuggaucCca...............   5      1   seq
......................................................................uaucacagaccgcuugCaucaca...............   12     1   seq
......................................................................uaucCcagaccgcuuggaucaca...............   4      1   seq
......................................................................uaucacagacGgcuuggaucaca...............   15     1   seq
......................................................................uaucacagaccgcuuggaucacG...............   370    1   seq
......................................................................Naucacagaccgcuuggaucaca...............   49     1   seq
......................................................................uaucacagaGcgcuuggaucaca...............   7      1   seq
......................................................................uauUacagaccgcuuggaucaca...............   105    1   seq
......................................................................uaucacagaccgcuuggaucacN...............   4      1   seq
......................................................................Aaucacagaccgcuuggaucaca...............   731    1   seq
......................................................................uaucacagaccgcuAggaucaca...............   83     1   seq
......................................................................uaucacagaccgcuuUgaucaca...............   31     1   seq
......................................................................uaucacagaNcgcuuggaucaca...............   2      1   seq
......................................................................uaucacagaccgcuuggaucaAa...............   42     1   seq
......................................................................uaucacagaccgcuuggaCcaca...............   42     1   seq
......................................................................uaucacagaccgcuuggauUaca...............   105    1   seq
```

Star **Mature**

```
cugccuccuucaccacccccuccugugcauggcucaccgcggucucagcgucuguggugcgcugaauucguaucacagaccgcuuggaucacagugcgcuguugugau
.....................................................................uaucacaAaccgcuuggaucaca...............	25	1	seq
.....................................................................uaucacagaccgcuuggaucaca...............	211562	0	seq
.....................................................................uaucacagaccgcuuggauAaca...............	87	1	seq
.....................................................................uaucacagaccgcuGggaucaca...............	24	1	seq
.....................................................................uaucacagaccgcGuggaucaca...............	11	1	seq
.....................................................................uaucUcagaccgcuuggaucaca...............	15	1	seq
.....................................................................uaucacagaccgcuugAaucaca...............	26	1	seq
.....................................................................uaucacagaccgcAuggaucaca...............	26	1	seq
.....................................................................uaucacagaccgAuuggaucaca...............	41	1	seq
.....................................................................uaucacagaccgcuuggaucacaC..............	5	1	seq
.....................................................................uaucacagaccgcuuggaucacag..............	1	0	seq
.....................................................................uaucacagaccgcuuggaucacUg..............	1	1	seq
.....................................................................uaucacagaccgcuuggaucacaA..............	403	1	seq
.....................................................................uaucacagaccgcuuggaucacaU..............	511	1	seq
.....................................................................uaucacagaccgcuuggaucacaUu.............	1420	1	seq
.....................................................................uaucacagaccgcuuggaucacagu.............	10	0	seq
.....................................................................uaucacagaccgcuuggaucacaCu.............	2	1	seq
.....................................................................uaucacagaccgcuuggaucacaAu.............	250	1	seq
.....................................................................uaucacagaccgcuuggaucacagA.............	1	1	seq
......................................................................aucacagaccgcuuggaucac................	15	0	seq
......................................................................Uucacagaccgcuuggaucaca...............	1	1	seq
......................................................................aucacagaccgcuuggaucaca...............	21	0	seq
......................................................................aucacagaccgcuuggaucacU...............	6	1	seq
......................................................................aucacagaccgcuuggaucacaU..............	11	1	seq
.......................................................................ucacagaccgcuuggaucaAa...............	1	1	seq
.......................................................................ucacagaccgcuuggaucaca...............	89	0	seq
.......................................................................ucacagaccgcuuggaucacagu.............	27	0	seq
.......................................................................ucacagaccgcuuAgaucacagu.............	1	1	seq
........................................................................cacagaccgcuuggaucaca...............	1	0	seq
........................................................................Uacagaccgcuuggaucaca...............	1	1	seq
.........................................................................acagaccgcuuggaucac................	6	0	seq
.........................................................................acagaccgcuuggaucaca...............	4	0	seq
..........................................................................cagaccgcuuggaucaca...............	1	0	seq
```
